# Plant-endophytes interaction influences the secondary metabolism in *Echinacea purpurea* (L.) Moench: an *in vitro* model

**DOI:** 10.1038/s41598-017-17110-w

**Published:** 2017-12-05

**Authors:** Valentina Maggini, Marinella De Leo, Alessio Mengoni, Eugenia Rosaria Gallo, Elisangela Miceli, Rose Vanessa Bandeira Reidel, Sauro Biffi, Luisa Pistelli, Renato Fani, Fabio Firenzuoli, Patrizia Bogani

**Affiliations:** 1 Department of Biology, University of Florence, Via Madonna del Piano 6, 50019 Sesto Fiorentino, Italy; 2 Department of Experimental and Clinical Medicine, University of Florence, Largo Brambilla 3, 50134 Florence, Italy; 3 Referring Center for Phytotherapy, Tuscany Region, Careggi University Hospital, Largo Brambilla 3, 50134 Florence, Italy; 4 Department of Pharmacy, University of Pisa, Via Bonanno 33, 56126 Pisa, Italy; 5 Botanical Garden Casola Valsenio, Via del Corso 6, 48010 Ravenna, Italy

## Abstract

The influence of the interaction(s) between the medicinal plant *Echinacea purpurea* (L.) Moench and its endophytic communities on the production of alkamides is investigated. To mimic the *in vivo* conditions, we have set up an infection model of axenic *in vitro E. purpurea* plants inoculated with a pool of bacterial strains isolated from the *E. purpurea* stems and leaves. Here we show different alkamide levels between control (not-inoculated) and inoculated plants, suggesting that the alkamide biosynthesis may be modulated by the bacterial infection. Then, we have analysed the branched-chain amino acids (BCCA) decarboxylase gene (GenBank Accession #LT593930; the enzymatic source for the amine moiety formation of the alkamides) expression patterns. The expression profile shows a higher expression level in the inoculated *E. purpurea* tissues than in the control ones. These results suggest that the plant-endophyte interaction can influence plant secondary metabolism affecting the therapeutic properties of *E. purpurea*.

## Introduction

*Echinacea purpurea* (L.) Moench (Asteraceae) is a medicinal plant with immune-modulatory and anti-inflammatory properties, whose roots and aerial parts are frequently used in Europe and North America for the preparation of therapeutic extracts for common cold^1^. It is rich in various phytochemicals including caffeic acid derivatives, alkamides and polysaccharides^2^. The concentrations of these bioactive compounds are species-specific and they may vary due to several factors such as plant material, cultivation techniques, plant tissue treatment, extraction methods and phytosanitary status^3^. Recently, the attention has been focused on the plant microbiota and its role in the production of secondary metabolites^4,5^. Many studies aim to investigate the influence of endophytic fungi on the production of plant bioactive molecules^6,7^ but the interest for the bacterial endophytes is considerably increasing^8^. Genomics and proteomics approaches have been applied to deepen the understanding of the plant-endophyte interaction^9^. Differential protein accumulations have been revealed in the proteome of *in vitro-*grown *Zea mays* ^10^ and Chinese hybrid poplar clone 741^11^ inoculated or not-inoculated with *Herbaspirillum seropedicae* and *Paenibacillus* sp., respectively. In particular, the role of the endophytes is investigated related to agricultural aspects (*e.g*. plant-growth promoting and biocontrol) and very few studies have been conducted on medicinal plants^3,12^. *E. purpurea* root extracts are reported to stimulate macrophage TNF-α production but the extracts obtained from *in vitro*-grown axenic *E. purpurea* do not induce the same result, supporting the hypothesis that it is originated from the interaction with bacterial endophytes^12^. Our previous study shows that different compartments of *E. purpurea*, namely stems and leaves (SL), roots (R) and the rhizosphere (RS), share very few strains^13^. This finding has been shown to be related to the antagonism existing between strains inhabiting the different compartments^14^ and to the degree of resistance to antibiotics^15^. Moreover, the presence of distinct bacterial communities in plant compartments could account for the different bioactive compounds found in the various plant organs. Therefore, *E. purpurea* represents an interesting and useful *in vitro* model for plant-bacterial interaction studies on the production of pharmacological relevant secondary metabolites as the alkamides.

Alkamides (alkylamides; fatty acid amides) are lipophilic compounds chemically composed of two moieties, an amine moiety acylated by a fatty acid-derived one^16^. They are important bioactive compounds dissimilarly distributed in the different compartments of *E. purpurea* ^17^. Indeed, a notable difference is reported for the total alkamide content between aerial parts and roots, which is mainly due to a larger presence of non-tetraene alkamides in roots than in other plant organs^18^. The main *Echinacea* spp. alkamides are the isomeric dodeca-2*E*, 4*E*, 8*Z*, 10*E*/*Z*-tetraenoic acid isobutylamides^19^. These compounds increase the TNF mRNA expression in macrophages and monocytes binding the cannabinoid CB2 receptor^20^. Furthermore, the alkylamides decrease mitogens-induced interleukin-2 secretion in Jurkat-T cells^21^ and show an *in vitro* inhibitory activity of the 5-lipoxygenase^22^ and the cyclooxygenase-1 and 2 enzymes^23^.

Recently, a pyridoxal phosphate-dependent (PLP) decarboxylating enzyme belonging to the Class II tryptophan synthase family that utilizes branched-chain amino acids (BCAA) as substrate has been suggested^24^. Isotope labelling analyses have revealed the generation of isobutylamine and 2-methylbutylamine (*i.e*. the amine moiety of the *E. purpurea* alkamides) from valine and isoleucine, respectively. PLP decarboxylase-like proteins have been identified in the proteome of *E. purpurea* through *in silico* analyses and their transcript levels correlated with alkamide accumulation patterns in *E. purpurea* tissues. Then, a valine decarboxylase (VDC), potentially involved in the generation of the amine moieties of the alkamides, has been identified.

**Figure 1:**
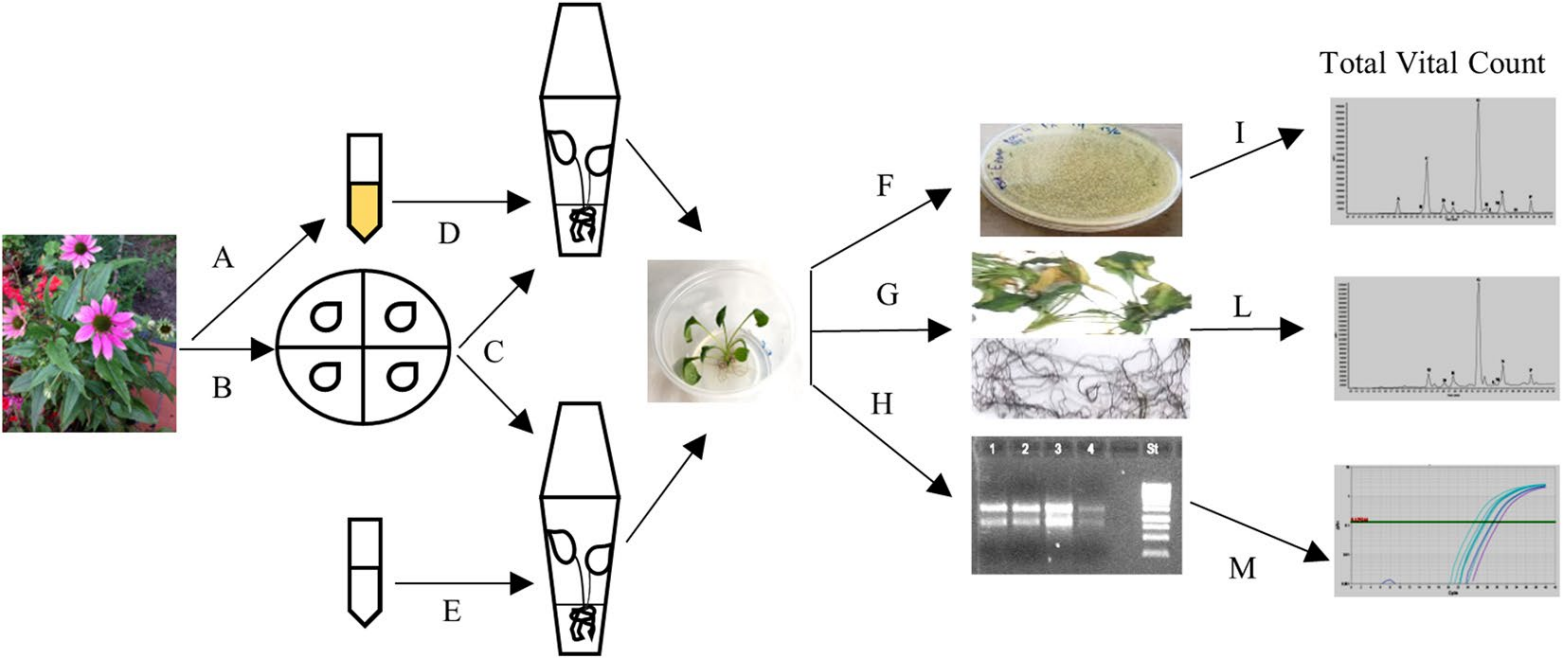
*In vitro* model system setting up to study the interaction between *Echinacea purpurea* plants and their stem/leaves endophytic bacteria. (**A)** Bacterial endophytes were isolated from the aerial compartment of *E*. *purpurea* plants. (**B)** *E*. *purpurea* seeds were provided by the common garden at the “Il Giardino delle Erbe”, Casola Valsenio, Italy and surface-sterilized. (**C)** Seeds were germinated in De Wit Culture tubes containing 5 ml of Linsmaier & Skoog Medium (LS) including vitamins. After root formation, the seedlings were transferred in Wavin flasks containing 50 ml of LS solid medium, supplemented with 3% sucrose and maintained in a plant growth chamber for a photoperiod of 16 h light a day. (**D,E**) After about 2 months, five *E*. *purpurea* plants were inoculated with 8 × 106 bacterial endophytes isolated from SL compartment of *E*. *purpurea* plants (**D**); five plants were used as control and were inoculated with sterilized saline solution (**E**). After 45 days, SL and root (R) samples from control and infected plants, were collected separately and sterilized. (**F–H**) Samples were then, separated in different aliquots (R and SL pooled separately). (**I**) One aliquot of each tissue was immediately used for the *in planta* bacterial growth analysis. (**L**) One aliquot of each tissue was weighed and dried at 60 °C to be used for *n*-hexane extracts preparation. (**M**) One aliquot of each tissue was ground to a fine powder in liquid nitrogen and successively used for RNA extraction.

The aim of this work is to check the involvement of the endophytic communities of *E. purpurea* plants in the regulation of bioactive compound (alkamide) accumulation. To this purpose, we set up an *in vitro* model system in which axenic *E. purpurea* plants, *in vitro* germinated from sterilized seeds, are inoculated with a pool of selected endophytes previously isolated from the aerial compartment of *E. purpurea* plants cultivated in open field (Fig. 1). We have evaluated the biochemical alkamide profiles estimating different alkamide levels in control (not-inoculated) and inoculated plants. We have also found that the level of *VDC* gene expression is higher in the inoculated *E. purpurea* tissues than in the control ones, establishing a close relationship between the endophyte presence and alkamide levels.

## Results

### Bacterial endophytes tend to re-colonize the native niche during plant infection

A pool of thirty-seven bacterial strains (Supplementary Table 1), isolated from the SL compartment of *E. purpurea* plants, was used to inoculate five axenic *in vitro* 2-months old *E. purpurea* plants each with 6 or 7 leaflets; five plants of the same age, used as control, were inoculated with sterilized saline solution. The infection experiment was repeated three times. Forty-five days after the infection, plants were analysed for bacterial colonization estimating the total viable count (TVC) as Colony Forming Units (CFU)/g into the host R and SL tissues. Data obtained revealed that the highest CFU/g was detected in the SL compartment (7.06 ± 6.50 log CFU/g), and the lowest one in the roots of infected plants (6.70 ± 5.82 log CFU/g; *P* < 0.001). This finding could indicate that the endophytes tended to re-colonize the native niche (SL compartment). The absence of bacteria in the control plant tissues and in the washing solutions confirmed the use of an axenic plant model and a successful sterilization procedure, respectively.

### Alkamide profiling analysis in different organs of control and infected plants

**Figure 2:**
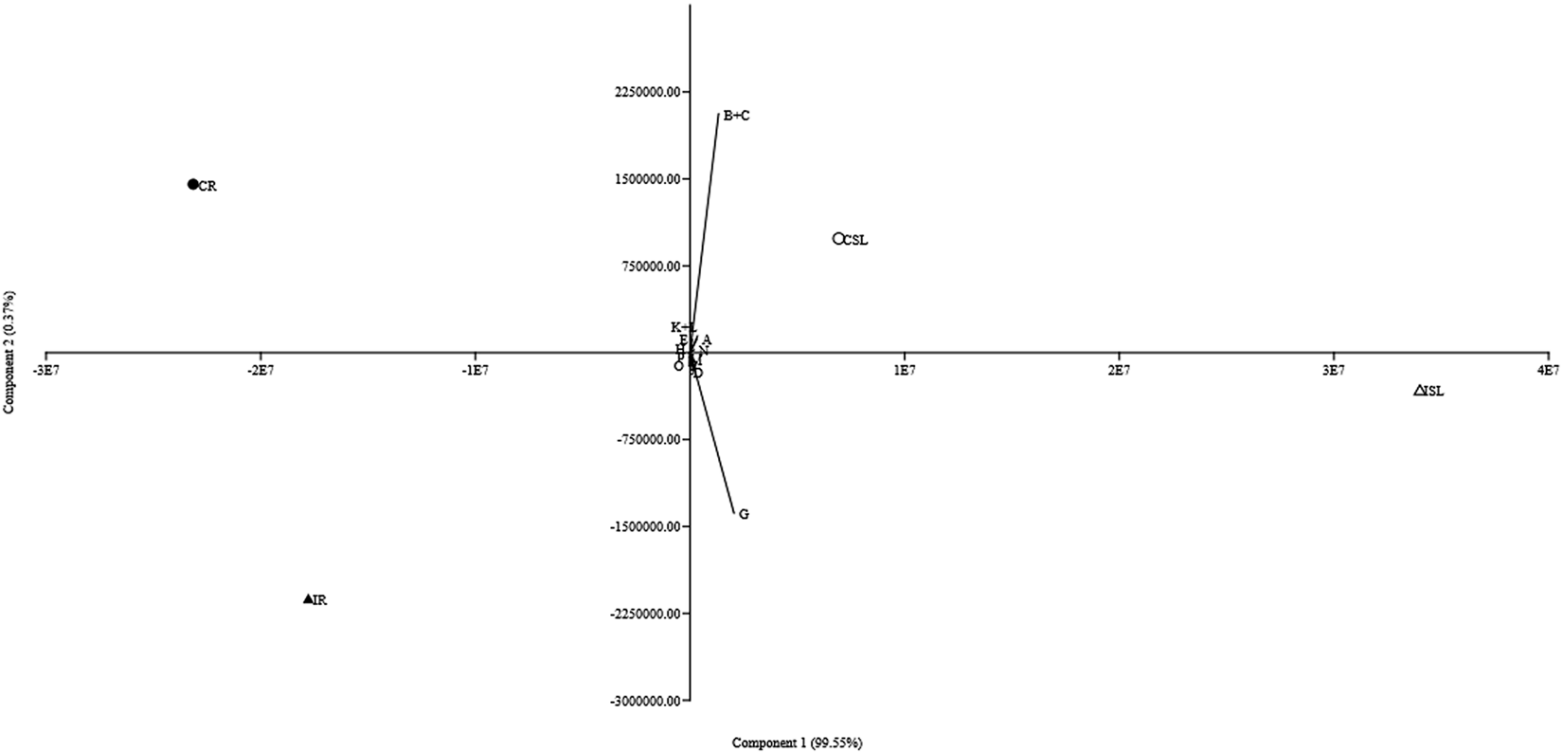
Principal Component Analysis of alkamide relative estimations of the four different *Echinacea purpurea* extracts. Letters on vectors indicate the HPLC peaks accounting for the differentiation of samples (see text for details). CSL, stem/leaves extract from control plants; ISL, stem/leaves extract from infected plants, CR, root extract from control plants; IR: root extract from infected plants.

The alkamide profiles of both control and infected R and SL extracts of *E. purpurea* pooled plants were investigated by means of high performance liquid chromatography (HPLC) coupled to a photo diode array (PDA)/ultraviolet (UV) detector and electrospray ionization tandem mass spectrometry (ESI-MS/MS). The LC-PDA/UV chromatograms of both *E. purpurea* R and SL extracts showed different alkamide profiles (Supplementary Figs 1 and 2). In particular, the SL extracts appeared to be richer in alkamide content, with twelve identified compounds (**1**-**12**, Table 1), respect to the R samples (compounds **5**, **7**-**12**, Table 1) even though for the peaks **A**, **B**, **G**, **K**, and **O** two isomers can occur and their exact identification was not possible based on MS fragmentation pathway. Some alkamides present in the SL extract (peaks **A**, **B**, **C**, **E**, **F**, **H**, **I**, and **M**) were not found in the R one. On the other hand, two R extracts showed the presence of the alkamides **13**/**14** (peak **O**) and **15** (peak **P**), which were absent in the SL ones. In all R and SL samples, the most representative alkamides were a mixture of the two co-eluting isomers dodeca-2*E*,4*E*,8*Z*,10*Z*-tetraenoic acid isobutylamide (**7**) and dodeca-2*E*,4*E*,8*Z*,10*E* tetraenoic acid isobutylamide (**8**). During the alkamide chromatography of both R and SL extracts, polyacetylene amides (peaks **A**, **B**, **D**, **O**, and **P**) elute early, followed by tetraenes (peaks **G** and **K**), trienes (peak **L**), and finally dienes (**N**). Interestingly, the analysis of the alkamide content in the *E. purpurea* plant organs also revealed the presence of seven alkamides not previously reported (peaks **C**, **E**, **F**, **H, I, J**, and **M**), but ascribable to the alkamide class due to the characteristic absorbance at 260 nm and typical ESI-MS and MS/MS spectra (Supplementary Table 3). A tentative level estimation of the main alkamides was performed on control and infected R and SL extracts by measuring the peaks area and calculating the mean of the replicates (Supplementary Table 4). Data were submitted to Principal Component Analysis (PCA), and the result was reported in Fig. 2: the vectors accounting for the R extracts were differentially oriented than those of the SL ones (F = 10.42; *P* < 0.001). In particular, the R samples presented C12 diene-diyne alkamides (peak **O** and **P**), whilst the SL samples contained C11 diene-diyne alkamides (peak **A** and **B**). Both samples showed a prevalence of C12 tetraene alkamides (peak **G** and **K**). Furthermore, PCA showed a discrimination among control and infected samples both for R and SL extracts and suggested that such differences were mainly created by the amount of the most abundant alkamide isomers **7** and **8** (peak **G**). The comparison of means (Supplementary Table 4) revealed that the amount of these alkamide isomers was significantly different for all samples (Tukey HSD *P* < 0.001). A relative estimation of the alkamide isomers **7** and **8** in the R and SL infected samples was also performed in respect to the levels of the relative control samples showing that the infection resulted in an increase of alkamide levels of about 70% in SL and 87% in R infected samples compared to the controls.

**Table 1:** Identification of alkamides detected in roots (R) and stem/leaves (SL) of control and infected *E. purpurea* plants. Compound numbers are referred to peaks in the chromatograms of Supplementary Figs 1 and 2. Peaks **C**, **E**, **F**, **H**, **I**, **J**, and **M** remained unidentified.

Compound	Peak^a^	Organ	Alkamides
**1**	**A**	SL	undeca-2*E*,4*Z*-diene-8,10-diynoic acid isobutylamide
**2**	**A**	SL	undeca-2*Z*,4*E*-diene-8,10-diynoic acid isobutylamide
**3**	**B**	SL	undeca-2*E*,4*Z*-diene-8,10-diynoic acid methylbutylamide
**4**	**B**	SL	undeca-2*Z*,4*E*-diene-8,10-diynoic acid methylbutylamide
**5**	**D**	R, SL	trideca-2*E*,7*Z*-diene-8,10-diynoic acid isobutylamide
**6**	**D**	SL	dodeca-2*E*,4*Z*,10*E*-triene-8-ynoic acid isobutylamide
**7**	**G**	R, SL	dodeca-2*E*,4*E*,8*Z*,10*Z*-tetraenoic acid isobutylamide
**8**	**G**	R, SL	dodeca-2*E*,4*E*,8*Z*,10*E* tetraenoic acid isobutylamide
**9**	**K**	R, SL	dodeca-2*E*,4*E*,8*Z*,10*Z*-tetraenoic acid methylbutylamide
**10**	**K**	R, SL	dodeca-2*E*,4*E*,8*Z*,10*E*-tetraenoic acid methylbutylamide
**11**	**L**	R, SL	dodeca-2*E*,4*E*,8*Z*-trienoic acid isobutylamide
**12**	**N**	R, SL	dodeca-2*E*,4*E*-dienoic acid isobutylamide
**13**	**O**	R	dodeca-2*E*,4*Z*-diene-8,10-diynoic acid isobutylbutylamide
**14**	**O**	R	dodeca-2*Z*,4*E*-diene-8,10-diynoic acid isobutylbutylamide
**15**	**P**	R	dodeca-2*E*,4*Z*-diene-8,10-diynoic acid 2-methylbutylamide

^a^Two alkamide isomers are present under the same peak: **A** (**1**, **2**), **B** (**3**, **4**), **G** (**7**, **8**), **K** (**9**, **10**), and **O** (**13**, **14**). The peak **D** is generated by the coelution of two not isomer alkamides (**5**, **6**). The *E*/*Z* stereochemistry is indicated in accordance with the literature^3,26–28^.

### BCCA decarboxylases genetic expression in different organs of control and infected plants

**Figure 3:**
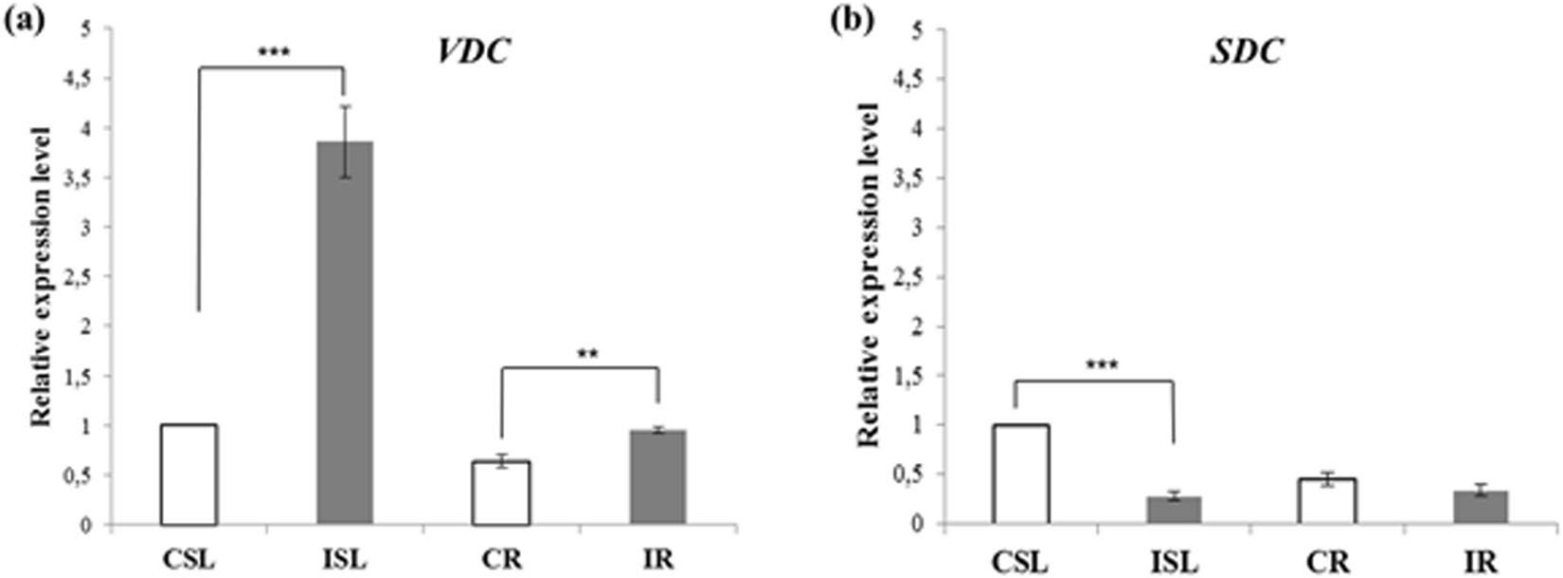
Gene expression of (**a**) valine decarboxylase (*VDC*) and (**b**) serine decarboxylase (S*DC*) in control and infected samples of *Echinacea purpurea* plants. Expression levels were normalized to expression in CSL. Data report average from three independent experiments (with 3 technical replicates each). Error bars: standard error of the mean (s.e.m.). Comparison between infected samples and the relative controls was determined by 2-tailed t-test (*P < 0.05, **P < 0.01, ***P < 0.001). CSL, stem/leaves extract from control plants; ISL, stem/leaves extract from infected plants, CR, root extract from control plants; IR: root extract from infected plants.

The relative quantification of *VDC* gene expression in the SL and R tissues of the control and infected *E. purpurea* plants was calculated in respect to the ubiquitin E2 (*UbE2*) gene expression in the three biological replicates. The amplification efficiency was optimal as achievable by the R^2^ and slope values (slope_*VDC*_ = –3.113; R^2^_*VDC*_ = 0.904; slope_*UbE2*_ = –3.182; R^2^_*UbE2*_ = 0.988). As depicted in Fig. 3a, the *VDC* gene expression levels were higher in the SL samples than the R ones (*P* < 0.01). The *VDC* transcription level in the infected SL samples was about 4 times more than in the SL control tissues (*P* < 0.001). Also, the expression level resulted increased in R infected tissues respect to the relative controls even if at a minor extent (*P* < 0.01). In parallel, the involvement of an enzyme (serine decarboxylase SDC, GenBank Accession #LT593931.1) not decarboxylating valine or isoleucine (to form isobutylamine or 2-methylbutylamine, the amine moieties of the *E. purpurea* alkamides) but utilizing the serine as substrate to generate ethanolamine^24^ was evaluated. The transcription of the *SDC* gene (slope_*SDC*_ = −3.135; R^2^_*SDC*_ = 0.982) appeared down-regulated in the infected SL samples respect to the SL control ones whilst the *SDC* expression levels were similar between control and infected R samples (Fig. 3b).

## Discussion

In this work we set up an *in vitro* system to study the influence of the interaction between bacterial endophytes and *E. purpurea* plants on the production of plant bioactive compounds.

The infection data revealed the bacterial tendency to reach their niche of origin passing through the R apparatus since a significant number of bacteria was detected also in the R tissues of the infected plants. This was in agreement with the current literature, according to which one of the ways used by endophytes to reach the aerial parts of the host plant was their migration through the xylem vascular system^25^ once that they have entered the plant roots. Hence, we speculated that all the endophytes could reach the SL compartment. However, we were not able to consider a post-infection time longer than 45 days since it would have induced several plant damages. Therefore, our data confirmed the hypothesis that differences in bacterial strains distribution between R and SL of *E. purpurea* could be related to the physiological conditions existing in the different plant tissues and organs representing a specific ecological niche, as previously suggested^13^.

On the basis of data obtained in this work, the R and SL extracts displayed different alkamide profiles, with twelve and ten compounds, respectively identified comparing their HPLC elution orders, ESI-MS/MS, and PDA/UV spectra with data reported in the literature^3,26–29^. The most abundant alkamides were the 2,4-diene type compounds and the two isomers dodeca-2*E*,4*E*,8*Z*,10*Z*-tetraenoic acid isobutylamide (**7**) and dodeca-2*E*,4*E*,8*Z*,10*E* tetraenoic acid isobutylamide (**8**) being predominant, according to previous studies performed on *in vivo* plants^30,31^. Moreover, seven alkamides (peaks **C**, **E**, **F**, **H**, **I**, **J**, and **M**) remained not identified, and among these one alkamide (peak **F**) was present only in the infected SL compartment. To our knowledge, these alkamides were not previously reported in *E. purpurea*.

The estimated total alkamide level was higher in the SL samples than in R ones, apparently in contrast with the literature data reporting that the *E. purpurea* roots contain more alkamides than the leaves^2^. However, the alkamide content in SL samples could be higher than the reported leaves amount since it was the sum of the leaves and the vegetative stems. In fact, in line with the literature^32^, the R and SL extracts were differentiated by the presence of the C12 and C11 diene-diyne alkamides (mainly reported in the stem), respectively. On the other hand, Qu *et al*.^18^ reported that the tetraene alkamides **7** and **8** accounted for the 75% in the aerial part and only for the 9% in the roots of *E. purpurea* plants cultivated into a field and these estimates resulted discordant with our results (about 50 and 70%, respectively). To this concern, one possible explanation could be that, to the best of our knowledge, this study is the first to estimate the *E. purpurea* alkamide level in an *in vitro* model whose experimental conditions could influence plant genetics and/or biochemical synthesis. However, the estimated alkamide level was higher in both infected R and SL, with a relative increase of alkamides **7** and **8** about 87% and 70% respectively, when compared to the controls, suggesting that the alkamide biosynthesis was modulated by the *E*. *purpurea* endophyte infection.

Concerning the *VDC* gene expression profiles, the highest expression level was detected in the infected *E*. *purpurea* tissues. By comparing results concerning both the chemical profiling analysis and the *VDC* gene expression data, it could be observed that the use of the proposed *in vitro* infection model system allowed us to demonstrate that the infection of *E. purpurea* axenic plants with their endophytes influenced the alkamide levels. In fact, both alkamide content and *VDC* transcription level resulted increased after the infection. The transcriptional up-regulation in the infected R samples was lower than the expected one: this result could be explained considering that the increase of the alkamide quantity in the infected R might be due to the transcriptional regulation of other enzymes required for the biosynthesis of the alkamide ammine and fatty acid moieties^24^. Interestingly, the SDC enzyme did not seem to contribute to the increase of alkamide levels confirming the specificity of the enzymatic source for the amine moiety formation of the alkamides (*i.e*. the *VDC* decarboxylase) as reported by Rizhsky *et al*.^24^.

The main objective of this work was to develop an *in vitro* model to study the role of the interaction between *E. purpurea* and its endophytes in the modulation of the plant secondary metabolism. Bacterial communities differed substantially between *E. purpurea* organs^13^ probably exerting a host selectivity able to modulate the community structure as reported for endophytic fungi^33^. In fact, the *E. purpurea* endophytes inoculated in axenic plants tended to re-colonize the native niche whose specific properties probably were influenced in turn by the natural endophytes. Chemical profiles and *VDC* genetic expression resulted quantitatively different between the control and infected plants, in particular in the SL compartment. Therefore, despite the *in vivo* status during bacterium-host interactions was difficult to mimic *in vitro*, the infection with *E. purpurea* SL endophytes modulated the characteristics resulting in axenic conditions, at least to some extent.

Consequently, the whole body of data obtained in this work strongly suggested that the secondary metabolism in *E. purpurea* was influenced by the plant-endophyte interaction thus possibly contributing to the therapeutic properties of this medicinal plant.

## Materials and Methods

### Bacterial cultures and plant material

Bacterial endophytes were isolated from the aerial compartment (stem and leaves) of *E. purpurea* plants grown at the “Il Giardino delle Erbe”, Casola Valsenio, Italy, as previously reported^13^. Stock cultures were grown at 30 °C on tryptone soy agar (TSA; Bio-Rad, USA) solid medium or tryptone soy broth (TSB, Bio-Rad, USA) liquid medium. *E. purpurea* seeds were provided by the “Il Giardino delle Erbe”.

### Seed sterilization and plating

Seeds were surface sterilized in order to prevent any unwanted fungal or bacterial growth. Seeds were immersed in a 70% (v/v) ethanol for one minute and, subsequently, in a 5% sodium hypochlorite solution for eight minutes. They were then rinsed three times with sterile distilled water, kept overnight at 4 °C in the dark for growth synchronization and then germinated in De Wit Culture tubes (LAB Associates BV, The Netherlands) containing 5 ml of Linsmaier & Skoog Medium (LS) including vitamins (Duchefa Biochemie, The Netherlands) at 24 ± 1 °C in the dark. After root formation, the seedlings were transferred in Wavin flasks (LAB Associates BV, The Netherlands) containing 50 ml of LS solid medium, supplemented with 3% sucrose, for a photoperiod of 16 h light a day for a minimum of two months. In order to validate the sterility of the obtained model system, cultivable endophyte multiplication into host tissues was checked: both shoots and roots were separately collected, washed in saline solution (0,9% NaCl, washing solution), surface sterilized in 1% (v/v) hypochlorite for 8 min and rinsed three times with sterile distilled water. Both samples were homogenized in saline solution and five replications of 100 μl of the homogenates were plated on TSA medium. Bacterial growth was scored after three days of plate incubation at 30 °C.

### Plant infection

*Inocula* of bacterial endophytes, isolated from SL compartment of *E. purpurea* plants, were incubated for three days at 30 °C in horizontal position and in agitation. The bacterial suspensions were then adjusted to 8 × 10^8^ CFU/ml (OD_600_ = 1). The optical density (OD) was measured in a biophotometer (Eppendorf, Germany). The pool generated from 100 μl of each diluted 1:10 OD_600_ suspension cultures was then centrifuged at 4000 rpm for 20 minutes and the pellet suspended in a correspondent volume of 0.9% saline solution. Five 2-months old *E. purpurea* plants were infected with 100 μl of bacterial suspension culture. Five plants were used as control and were infected with 100 μl of sterilized saline solution. Plants were then incubated in the growth chamber at 24 ± 1 °C. After 45 days, SL and R samples from control and infected plants, were collected separately, firstly washed in saline solution and then sterilized in 1% (v/v) hypochlorite for 8 min. Then, both tissue samples were washed three times with sterile distilled water and separated in different aliquots. R and SL aliquots were weighed and dried at 60 °C to be used for *n*-hexane extract preparation. R and SL aliquots of fresh material were ground to a fine powder in liquid nitrogen and successively stored at – 80 °C for RNA extraction. Finally, 1.0 g of fresh R and SL tissues were immediately used for the *in planta* bacterial growth analysis. The experiment was performed in triplicate.

### *In planta* bacterial growth analysis

In order to evaluate endophytes multiplication into host tissues, 1.0 g of each sample was homogenized in saline solution and 100 µl of the homogenate were serially diluted up to 10^−7^/ml cells. Five replications of each dilution were plated on TSA medium. The washing solution and the distilled water after the last wash were also diluted to check the presence of bacterial cells on the surface of the tissues and the outcome of the sterilization procedure. Bacterial growth was scored after two, three and four days of incubation of the plates at 30 °C.

### Sample preparation for HPLC analysis of alkamides

Dried and powdered R and SL of control and infected *E. purpurea* plants from three independent experiments were pooled and extracted at room temperature with *n*-hexane (1.0 g of dried drug in 30 ml of solvent for three times, every 24 h) as detailed in Supplementary Table 5. Solutions of each *n*-hexane residue from R and SL samples were then prepared dissolving the respective *n*-hexane extract in an opportune volume of methanol and then centrifuging the mixture. Finally, 20 μl of each supernatant solution (2.0 mg/ml) were injected for HPLC-PDA/UV-ESI-MS/MS analysis. The experiment was performed in duplicate.

### HPLC-PDA/UV-ESI-MS/MS analyses

Qualitative HPLC-PDA/UV-ESI-MS/MS analyses were performed using a Surveyor LC pump, a Surveyor autosampler, coupled with a Surveyor PDA detector, and a LCQ Advantage ion trap mass spectrometer (ThermoFinnigan) equipped with Xcalibur 3.1 software. Analyses were performed using a 4.6 × 250 mm, 4 µm, Synergi Fusion-RP column (Phenomenex). The eluent was a mixture of methanol (solvent A) and a 0.1% v/v aqueous solution of formic acid (solvent B). A linear gradient of increasing 55% to 85% A was developed within 45 min. The column was successively washed for 15 min with methanol and equilibrated with 55% A for 10 min. Elution was performed at a flow rate of 0.8 ml/min with a splitting system of 2:8 to MS detector (160 ml/min) and PDA detector (640 ml/min), respectively. The volume of the injected methanol solutions was 20 μl. Analyses were performed with an ESI interface in the positive mode. The ionization conditions were optimized and the parameters used were as follows: capillary temperature, 270 °C; capillary voltage, 29.0 V; tube lens offset, 50.0 V; sheath gas flow rate, 60.00 arbitrary units; auxiliary gas flow rate, 3.00 arbitrary units; spray voltage, 4.50 kV; scan range of *m/z* 150–1200. N_2_ was used as the sheath and auxiliary gas. PDA data were recorded with 200–600 nm range with preferential channel as the detection wavelength 260 nm.

### Quantitative real time PCR (qRT-PCR) analysis

Total RNA from R and SL was extracted from *E. purpurea* tissues using the RNeasy Micro Kit (Qiagen, USA) and quantified by Qubit® 2.0 fluorimeter. One μg of total RNA for each sample was reverse-transcribed using the Quantitect Reverse Transcription Kit according to the manufacturer’s instructions (Qiagen) including a treatment with 1X gDNA Wipeout Buffer to remove any remaining DNA. The relative abundance of the *E. purpurea* cDNA for BCAA decarboxylases (*VDC*, GenBank Accession #LT593930; *SDC*, GenBank Accession #LT593931.1) was determined on a QUANTSTUDIO 7 FLEX (Applied Biosystems, USA) using the QuantiNova SYBR Green PCR Kit (Qiagen, USA). Ubiquitin E2 genetic expression was used as internal reference to normalize mRNA content. The quantification of the expression was measured by the comparative Ct (2^−∆∆Ct^) method^34^. Target gene expression was relative to the control SL cDNA, which has been adopted as calibrator. The experiment was conducted in triplicate. Primer3 software^35^ was used to design primers specific to *SDC* template (epa_locus_952_iso_8_len_1801_ver_2) as available in the Medicinal Plant Genomics Resource (http://medicinalplantgenomics.msu.edu). The primer sequences for the *VDC* gene were reported in Rizhsky *et al*.^24^. All primers listed in Supplementary Table 2 were synthesized by Eurofins Genomics (Ebersberg, Germany). Independent RT-PCR products were sequenced to control for primer specificity.

### Statistical analyses

Means and standard deviations of the bacterial TVC data of the three biological replicates were estimated and compared by one-way analysis of variance between R and SL samples. To evaluate whether the level estimations of the alkamides (mean peak area values) were useful in reflecting the chemical relationships between R and SL samples (controls and infected ones), a PCA was performed^36^. One-way analysis of variance followed by Tukey test was used to compare peak area values between control and infected plants and to identify the alkamides mainly responsible of the differences between the samples. Comparison of the qRT-PCR data of the three biological replicates was determined by 2-tailed t-test. *P* < 0.05 was considered significant (*P < 0.05, **P < 0.01, ***P < 0.001). Error bars are shown as s.e.m. The analyses were performed by using the modules present in the PAST program, version 3.15^37^.

### Data availability

The Authors declare that all the data supporting the findings of this study are available within the manuscript and its Supplementary Material and from the Corresponding Author on request.

## Electronic supplementary material

Supplementary Figure 1

Supplementary Figure 2

Supplementary Table 1

Supplementary Table 2

Supplementary Table 3

Supplementary Table 4

Supplementary Table 5
