# Supplementary material for: Plant-endophytes interaction influences the secondary metabolism in Echinacea purpurea (L.) Moench: an in vitro model
Source: Sci Rep. 2017 Dec 5;7:16924. doi: 10.1038/s41598-017-17110-w (PMC5717142; doi:10.1038/s41598-017-17110-w)
Supplement: Supplementary file 3 — Supplementary Table 1 [file 41598_2017_17110_MOESM3_ESM.pdf]

Plant-endophytes interaction influences the secondary metabolism in *Echinacea purpurea* (L.) Moench: an *in vitro* model.

Valentina Maggini[1,2,3], Marinella De Leo[4], Alessio Mengoni[1], Eugenia Rosaria Gallo[2,3], Elisangela Miceli[1], Rose Vanessa Bandeira Reidel[4], Sauro Biffi[5], Luisa Pistelli[4], Renato Fani[1], Fabio Firenzuoli[3] and Patrizia Bogani[1]

[1]Department of Biology, University of Florence, Via Madonna del Piano 6, 50019 Sesto Fiorentino, Italy; [2]Department of Experimental and Clinical Medicine, University of Florence, Largo Brambilla 3, 50134 Florence, Italy; [3]Referring Center for Phytotherapy, Tuscany Region, Careggi University Hospital, Largo Brambilla 3, 50134 Florence, Italy; [4]Department of Pharmacy, University of Pisa, Via Bonanno 33, 56126 Pisa, Italy; [5]Botanical Garden Casola Valsenio, Via del Corso 6, 48010 Ravenna, Italy

Supplementary Table 1. The thirty-seven bacterial strains from *E. purpurea* used in this work.

Strain code	Genus	GenBank accession of partial 16S rRNA gene sequence
EpSL1	*Curtobacterium* sp.	KJ642423
EpSL2	*Curtobacterium* sp.	KJ642424
EpSL4	*Microbacterium* sp.	KJ642438
EpSL5	*Bacillus* sp.	KJ642422
EpSL16	*Arthrobacter* sp.	KJ642432
EpSL17	*Staphylococcus* sp.	KJ642469
EpSL18	*Arthrobacter* sp.	KJ642419
EpSL20	*Pseudomonas* sp.	KJ642444
EpSL22	*Staphylococcus* sp.	KJ642476
EpSL25	*Pseudomonas* sp.	KJ642442
EpSL27	*Arthrobacter* sp.	KJ642420
EpSL31	*Rhodobacter* sp.	KJ642453
EpSL32	*Sphingomonas* sp.	KJ642455
EpSL34	*Staphylococcus* sp.	KJ642465
EpSL35	*Arthrobacter* sp.	KJ642421
EpSL37	*Pseudomonas* sp.	KJ642443
EpSL39	*Pseudomonas* sp.	KJ642336
EpSL40	*Staphylococcus* sp.	KJ642466
EpSL43	*Pseudomonas* sp.	KJ642443
EpSL50	*Sphingomonas* sp.	KJ642457
EpSL54	*Frigoribacterium* sp.	KJ642425
EpSL59	*Frigoribacterium* sp.	KJ642426
EpSL62	*Staphylococcus* sp.	KJ642472
EpSL64	*Frigoribacterium* sp.	KJ642427
EpSL65	*Microbacterium* sp.	KJ642440
EpSL70	*Sphingomonas* sp.	KJ642460
EpSL80	*Frigoribacterium* sp.	KJ642428
EpSL81	*Agrococcus* sp.	KJ642418
EpSL82	*Methylobacterium* sp.	KJ642437
EpSL83	*Sphingomonas* sp.	KJ642462
EpSL84	*Frigoribacterium* sp.	KJ642430
EpSL87	*Kineococcus* sp.	KJ778698
EpSL89	*Staphylococcus* sp.	KJ642473
EpSL91	*Frigoribacterium* sp.	KJ642429
EpSL95	*Staphylococcus* sp.	KJ642470
EpSL96	*Staphylococcus* sp.	KJ642474
EpSL102	*Staphylococcus* sp.	KJ642464
