# Supplementary material for: Plant-endophytes interaction influences the secondary metabolism in Echinacea purpurea (L.) Moench: an in vitro model
Source: Sci Rep. 2017 Dec 5;7:16924. doi: 10.1038/s41598-017-17110-w (PMC5717142; doi:10.1038/s41598-017-17110-w)
Supplement: Supplementary file 4 — Supplementary Table 2 [file 41598_2017_17110_MOESM4_ESM.pdf]

Plant-endophytes interaction influences the secondary metabolism in *Echinacea purpurea* (L.) Moench: an *in vitro* model.

Valentina Maggini[1,2,3], Marinella De Leo[4], Alessio Mengoni[1], Eugenia Rosaria Gallo[2,3], Elisangela Miceli[1], Rose Vanessa Bandeira Reidel[4], Sauro Biffi[5], Luisa Pistelli[4], Renato Fani[1], Fabio Firenzuoli[3] and Patrizia Bogani[1]

[1]Department of Biology, University of Florence, Via Madonna del Piano 6, 50019 Sesto Fiorentino, Italy; [2]Department of Experimental and Clinical Medicine, University of Florence, Largo Brambilla 3, 50134 Florence, Italy; [3]Referring Center for Phytotherapy, Tuscany Region, Careggi University Hospital, Largo Brambilla 3, 50134 Florence, Italy; [4]Department of Pharmacy, University of Pisa, Via Bonanno 33, 56126 Pisa, Italy; [5]Botanical Garden Casola Valsenio, Via del Corso 6, 48010 Ravenna, Italy

Supplementary Table 2. DNA-primers used in qRT-PCR analysis.

mRNA fragment	Primer sequence	Reference
UbE2	5’-ATGTTGACCCGAGAATGGAA-3’	Rizhsky L *et al.*[24]
	5’-TGGAATGGATGATGGTGATG-3’	
VDC	5’-TGCTGTGTGCCTGCTTTAAC-3’	
	5’-CCTCCAGTGCCTGAAAACAT-3’	
SDC	5’- AAATGGTCATACGGGTCGCA-3’	This work
	5’-GGCCATAGATGTCATACTGGCC-3’	
