# Supplementary material for: Plant-endophytes interaction influences the secondary metabolism in Echinacea purpurea (L.) Moench: an in vitro model
Source: Sci Rep. 2017 Dec 5;7:16924. doi: 10.1038/s41598-017-17110-w (PMC5717142; doi:10.1038/s41598-017-17110-w)
Supplement: Supplementary file 5 — Supplementary Table 3 [file 41598_2017_17110_MOESM5_ESM.pdf]

Plant-endophytes interaction influences the secondary metabolism in *Echinacea purpurea* (L.) Moench: an *in vitro* model.

Valentina Maggini[1,2,3], Marinella De Leo[4], Alessio Mengoni[1], Eugenia Rosaria Gallo[2,3], Elisangela Miceli[1], Rose Vanessa Bandeira Reidel[4], Sauro Biffi[5], Luisa Pistelli[4], Renato Fani[1], Fabio Firenzuoli[3] and Patrizia Bogani[1]

[1]Department of Biology, University of Florence, Via Madonna del Piano 6, 50019 Sesto Fiorentino, Italy; [2]Department of Experimental and Clinical Medicine, University of Florence, Largo Brambilla 3, 50134 Florence, Italy; [3]Referring Center for Phytotherapy, Tuscany Region, Careggi University Hospital, Largo Brambilla 3, 50134 Florence, Italy; [4]Department of Pharmacy, University of Pisa, Via Bonanno 33, 56126 Pisa, Italy; [5]Botanical Garden Casola Valsenio, Via del Corso 6, 48010 Ravenna, Italy

Supplementary Table 3. Spectral (UV and ESI-MS/MS) and chromatographic data (retention time, t_R) of alkamides **1-15** detected in *n*-hexane extracts of *E. purpurea* not-infected stem/leaves (SL) and roots (R), used as control, and SL and R infected by *E. purpurea* SL endophytes. MS/MS data are obtained from the fragmentation of the $[M+H]^+$ precursor ions. The peaks are related to those reported in Supplementary Figs. 2 and 3. Compound numbers are referred to alkamides listed in Table 1.

Peak	Compound	t_R (min)	M	$[M+H]^+$	$[M+Na]^+$	$[2M+H]^+$	$[2M+Na]^+$	MS/MS base peak	MS/MS ions (*m/z*)	λ_{max} (nm)	Reference
**A**	**1** and /or **2**	18.7	229	230	252	459	481	131	174[a], 157[b], 146, 129[e], 116, 91	260	26
**B**	**3** and /or **4**	22.8	243	244	266	487	509	131	216, 174[c], 157[d], 129[f], 117, 91	260	29
**C**	NI	23.7	263	264	286	527	549	246	222, 175, 147, 105, 93	260	NR
**D**	**5**	26.3	257	258	280	515	537	202[a]	185[b], 159, 157[e], 143, 131, 117, 91	260	29
	**6** (only in leaves)	26.7	245	246	268	491	513	147	190[a], 173[b], 145, 131[e], 119, 105, 91	260	26
**E**	NI	28.3	265	266	288	531	553		–	260	NR
**F**	NI	30.1	245	246	268	491	513		221, 203, 190, 175, 173, 147, 119, 105, 91	260	NR
**G**	**7** and/or **8**	32.5	247	248	270	495	518	149	192[a] , 175[b], 147[e], 142, 93, 74	260	26
**H**	NI	33.9	267	268	290	535	557	–	–	260	NR
**I**	NI	34.7	285	286	308	571	593	230	213, 195, 185, 171, 145, 131, 117, 95	250	NR
**J**	NI	35.4	278	279	301	557	579	204	234, 223, 203, 181, 163, 149, 135, 95	260	NR
**K**	**9** or **10**	36.4	261	262	284	525	545	149	234, 204, 192[c], 181, 175[d], 156, 147[f], 133, 121, 107, 93	250	28
**L**	**11**	36.8	249	250	272	499	521	167	194[a], 177[b], 149[e], 135, 121, 109, 95	275	26
**M**	NI	39.0	275	276	298	551	573	177	220, 203, 175, 135, 121, 95		NR
**N**	**12**	41.6	251	252	274	503	525	179[b]	196[a], 161, 137, 151[e], 119, 95,79	260	26
**O**	**13** or **14**	23.5	243	244	266	487	509	145	216, 202, 188[a], 171[b], 143[e], 129, 117, 105, 91	260	29
**P**	**15**	27.7	257	258	280	515	537	145	230, 188[c], 171[d], 143[f], 117, 105, 91	260	29

[a]Fragment $[M+H-56]^+$ originated by the loss of the isobutyl group attached to the nitrogen; [b]fragment $[M+H-73]^+$ due to the loss of the isobutyl amine; [c]fragment $[M+H-70]^+$ due to the loss of the 2-methylbutyl group attached to the nitrogen; [d]fragment $[M+H-87]^+$ corresponding to the loss of the 2-methylbutyl amine; [e]fragment $[M+H-101]^+$ due to the loss of the amide portion from an isobutylamide; [f]fragment $[M+H-115]^+$ originated by the loss of the amide portion from a 2-methylbutylamide. NR: not reported; NI: not identified. Alkamides not previously reported (peaks **C**, **E**, **F**, **H, I, J** and **M**) are ascribable to the alkamide class due to the characteristic absorbance at 260 nm and typical ESI-MS and MS/MS spectra Indeed, masses of alkamide ions are detected with ESI-MS in the positive mode, originating protonated $[M+H]^+$ and sodiated molecules $[M+Na]^+$. Protonated $[2M+H]^+$ and sodiated dimers $[2M+Na]^+$ were also commonly observed. Alkamides occurring in *E. purpurea* had structures containing ethylenic and/or acetylenic bonds and an amide portion, constituted by an isobutylamide or a 2-methylbutylamide moiety[2], displaying a high absorbance at 260 nm. These alkamides were structurally similar and many of them were isomers difficult to separate. Electrospray ionization techniques combined with multi-stage tandem spectrometry (MS^n) had the advantage of discriminating isobutylamides and 2-methylbutylamides[25]. Furthermore, isomeric alkamides could be distinguished by MS^n fragmentation patterns[28]. For isobutylamides the MS/MS spectra of deprotonated molecules (compounds **1**/**2**, **5**-**8**, **11**, **12**, and **13**/**14**) showed fragments corresponding to the loss of 56 u for the isobutyl group and 73 u for the isobutyl amine. Fragments corresponding to the loss of 70 u (for the 2-methylbutyl group) and 87 u (2-methylbutyl amine) could be observed for 2-methylbutylamides (compounds **3**/**4**, **9**/**10**, and **15**). In addition, diagnostic fragments corresponding to the alkyl chains due to the cleavage of the amide portions were detected (101 u for isobutylamides and 115 u for 2-methylbutylamides)[26,29].
