# Supplementary material for: Plant-endophytes interaction influences the secondary metabolism in Echinacea purpurea (L.) Moench: an in vitro model
Source: Sci Rep. 2017 Dec 5;7:16924. doi: 10.1038/s41598-017-17110-w (PMC5717142; doi:10.1038/s41598-017-17110-w)
Supplement: Supplementary file 6 — Supplementary Table 4 [file 41598_2017_17110_MOESM6_ESM.pdf]

Plant-endophytes interaction influences the secondary metabolism in *Echinacea purpurea* (L.) Moench: an *in vitro* model.

Valentina Maggini[1,2,3], Marinella De Leo[4], Alessio Mengoni[1], Eugenia Rosaria Gallo[2,3], Elisangela Miceli[1], Rose Vanessa Bandeira Reidel[4], Sauro Biffi[5], Luisa Pistelli[4], Renato Fani[1], Fabio Firenzuoli[3] and Patrizia Bogani[1]

[1]Department of Biology, University of Florence, Via Madonna del Piano 6, 50019 Sesto Fiorentino, Italy; [2]Department of Experimental and Clinical Medicine, University of Florence, Largo Brambilla 3, 50134 Florence, Italy; [3]Referring Center for Phytotherapy, Tuscany Region, Careggi University Hospital, Largo Brambilla 3, 50134 Florence, Italy; [4]Department of Pharmacy, University of Pisa, Via Bonanno 33, 56126 Pisa, Italy; [5]Botanical Garden Casola Valsenio, Via del Corso 6, 48010 Ravenna, Italy

Supplementary Table 4. Average, standard deviation (SD) and percentage of abundance of the alkamides identified in the extracts from R and SL samples **(**CSL: stem/leaves extract from control plants; ISL: stem/leaves extract from infected plants; CR: root extract from control plants; IR: root extract from infected plants). Pairwise comparison (Tukey HSD) was performed between all the samples: different letters in the same row indicate significant differences (P<0.001).

	**Peak Area mean ± SD *10^6 (%)**			
	**C R**	**I R**	**C SL**	**I SL**
**A**			2.672 ± 0.022 (4.33)	7.124 ± 0.128 (6.26)
**B+C**			15.804 ± 0.151 (25.60)	28.915 ± 0.143 (25.41)
**D**	0.219 ± 0.007 (2.11)	0.530 ± 0.020 (3.01)	2.028 ± 0.018 (3.27)	4.842 ± 0.365 (4.26)
**E**			1.114 ± 0.034 (1.80)	2.350 ± 0.198 (2.06)
**F**				0.401 ± 0.105 (0.35)
**G**	7.435 ± 0.427 (71.35)^{a}	13.906 ± 0.098 (78.81)^{b}	32.494 ± 0.169 (52.64)^{c}	55.107 ± 0.050 (48.44)^{d}
**H**			0.394 ± 0.058 (0.64)	0.899 ± 0.008 (0.79)
**I**			0.055 ± 0.002 (0.09)	0.067± 0.006 (0.06)
**J**	0.028 ± 0.020 (0.27)	0.136 ± 0.011 (0.77)	0.001 ± 0.001 (0.01)	0.019 ± 0.010 (0.02)
**K+L**	1.236 ± 0.013 (11.86)	1.581 ± 0.013 (8.96)	4.996 ± 0.183 (8.09)	9.148 ± 0.090 (8.04)
**M**			0.042 ± 0.014 (0.07)	0.111 ± 0.040 (0.10)
**N**	0.418 ± 0.006 (4.01)	0.520 ± 0.373 (2.95)	2.133 ± 0.009 (3.46)	4.790 ± 0.099 (4.21)
**O**	0.707 ± 0.086 (6.79)	0.650 ± 0.026 (3.68)		
**P**	0.377 ± 0.022 (3.61)	0.321 ± 0.010 (1.82)		
