# Supplementary material for: Plant-endophytes interaction influences the secondary metabolism in Echinacea purpurea (L.) Moench: an in vitro model
Source: Sci Rep. 2017 Dec 5;7:16924. doi: 10.1038/s41598-017-17110-w (PMC5717142; doi:10.1038/s41598-017-17110-w)
Supplement: Supplementary file 7 — Supplementary Table 5 [file 41598_2017_17110_MOESM7_ESM.pdf]

Plant-endophytes interaction influences the secondary metabolism in *Echinacea purpurea* (L.) Moench: an *in vitro* model.

Valentina Maggini[1,2,3], Marinella De Leo[4], Alessio Mengoni[1], Eugenia Rosaria Gallo[2,3], Elisangela Miceli[1], Rose Vanessa Bandeira Reidel[4], Sauro Biffi[5], Luisa Pistelli[4], Renato Fani[1], Fabio Firenzuoli[3] and Patrizia Bogani[1]

[1]Department of Biology, University of Florence, Via Madonna del Piano 6, 50019 Sesto Fiorentino, Italy; [2]Department of Experimental and Clinical Medicine, University of Florence, Largo Brambilla 3, 50134 Florence, Italy; [3]Referring Center for Phytotherapy, Tuscany Region, Careggi University Hospital, Largo Brambilla 3, 50134 Florence, Italy; [4]Department of Pharmacy, University of Pisa, Via Bonanno 33, 56126 Pisa, Italy; [5]Botanical Garden Casola Valsenio, Via del Corso 6, 48010 Ravenna, Italy

Supplementary Table 5. Sample preparation for HPLC analysis of the alkamides in the extracts from R and SL samples **(**CSL: stem/leaves extract from control plants; ISL: stem/leaves extract from infected plants; CR: root extract from control plants; IR: root extract from infected plants).

**Sample**	**Fresh weight** (g)	**Dry weight** (g)	***n*-hexane extract** (mg)
**C R**	2.53	0.28	7.8
**I R**	1.90	0.24	2.4
**C SL**	5.90	1.26	17.4
**I SL**	4.12	0.92	11.2
